# Sense of Coherence and Mental Health in Pediatric Nurses

**DOI:** 10.1192/j.eurpsy.2026.11809

**Published:** 2026-07-31

**Authors:** M. Liakoura, I. Koutelekos, A. Soldatou, A. Zartaloudi

**Affiliations:** 1 Nurse, MSc in General Pediatrics and Pediatric Subspecialties - Clinical Practice and Research; 2 Department of Nursing, Associate Professor, University of West Attica; 3 Medical School, Professor, University of Athens, Athens, Greece

## Abstract

**Introduction:**

Pediatric nurses are exposed to high levels of occupational stress due to the complex physical, emotional, and psychosocial demands of caring for children and their families. The cumulative psychological burden may manifest as anxiety, depression, burnout, or even cynicism and hostility, while the Sense of Coherence (SOC) appears to function as a protective factor that enhances resilience and mental health.

**Objectives:**

This study aims to investigate the psychological burden of pediatric nurses and examine the role of Sense of Coherence in moderating stress, emotional exhaustion, and maladaptive coping patterns in demanding clinical environments.

**Methods:**

A narrative review of the literature was conducted, focusing on international and European studies addressing stress, anxiety, depression, emotional burden, burnout, and cynicism among pediatric nurses. Theoretical models, such as Antonovsky’s salutogenic model and Hochschild’s theory of emotional labor, were used as the conceptual framework. The selected articles were published in the PubMed database within the past decade.

**Results:**

Evidence indicates that pediatric nurses experience significantly higher levels of stress and mental health difficulties compared with the general nursing population, particularly in pediatric oncology and emergency care settings. Stronger SOC is associated with better coping, lower psychological morbidity, and reduced risk of professional burnout and cynicism. Lack of organizational support, understaffing, and inadequate resources exacerbate emotional strain, while trust, teamwork, and supportive leadership serve as protective factors.

**Conclusions:**

Pediatric nursing is one of the most mentally demanding specialties in healthcare. Enhancing the SOC of pediatric nurses through targeted interventions, organizational support, and psychosocial training can mitigate stress and promote mental health. Understanding these mechanisms is essential for designing effective mental health interventions and safeguarding workforce sustainability.

**Disclosure of Interest:**

None Declared

